# Post-Transplant Bone Disease in Kidney Transplant Recipients: Diagnosis and Management

**DOI:** 10.3390/ijms25031859

**Published:** 2024-02-03

**Authors:** Jia Wei Teh, Conall Mac Gearailt, David W. P. Lappin

**Affiliations:** 1 Department of Nephrology, Galway University Hospital, H91 YR71 Galway, Ireland; 2 Department of Rheumatology, Galway University Hospital, H91 YR71 Galway, Ireland; 3 School of Medicine, University of Galway, H91 TK33 Galway, Ireland

**Keywords:** kidney failure, kidney transplantation, post-transplant bone disease, osteoporosis, chronic kidney disease–mineral bone disease

## Abstract

Kidney transplantation is the preferred gold standard modality of treatment for kidney failure. Bone disease after kidney transplantation is highly prevalent in patients living with a kidney transplant and is associated with high rates of hip fractures. Fractures are associated with increased healthcare costs, morbidity and mortality. Post-transplant bone disease (PTBD) includes renal osteodystrophy, osteoporosis, osteonecrosis and bone fractures. PTBD is complex as it encompasses pre-existing chronic kidney disease–mineral bone disease and compounding factors after transplantation, including the use of immunosuppression and the development of de novo bone disease. After transplantation, the persistence of secondary and tertiary hyperparathyroidism, renal osteodystrophy, relative vitamin D deficiency and high levels of fibroblast growth factor-23 contribute to post-transplant bone disease. Risk assessment includes identifying both general risk factors and kidney-specific risk factors. Diagnosis is complex as the gold standard bone biopsy with double-tetracycline labelling to diagnose the PTBD subtype is not always readily available. Therefore, alternative diagnostic tools may be used to aid its diagnosis. Both non-pharmacological and pharmacological therapy can be employed to treat PTBD. In this review, we will discuss pathophysiology, risk assessment, diagnosis and management strategies to manage PTBD after kidney transplantation.

## 1. Introduction

Chronic kidney disease (CKD) is defined as abnormalities in kidney structure or function, present for over 3 months, with implications for health. CKD is classified based on cause, glomerular filtration category and albuminuria category according to Kidney Disease Improving Global Outcomes (KDIGO) [1]. Kidney failure or end-stage kidney disease can progress from CKD. The incidence rates of kidney failure have remained stable from 2003 to 2016 in high-income countries but have risen in East and Southeast Asia [2]. According to the United States Renal Data System (USRDS), the incidence rate of kidney failure was 363 cases per million persons, of which 83.9% were treated with in-centre haemodialysis, 12.7% with peritoneal dialysis, 0.3% with home haemodialysis, and 3.1% with pre-emptive kidney transplantation as their incident kidney replacement therapy [3]. In 2020, the prevalence of kidney failure was 2271 cases per million persons in the United States [3].

Kidney transplantation is the preferred gold-standard modality of treatment for kidney failure as it offers long-term benefits, including the best survival and quality of life for patients [4,5]. Bone disease after kidney transplantation is highly prevalent in patients living with a kidney transplant and is associated with high rates of hip fractures, which are associated with increased healthcare costs, morbidity and mortality [6]. Post-transplant bone disease (PTBD) is an all-encompassing term used to describe a variety of bone diseases diagnosed after kidney transplantation, including renal osteodystrophy, osteoporosis, bone fracture and osteonecrosis [7]. Kidney transplantation does not completely reverse chronic kidney disease—mineral bone disease (CKD-MBD) [8]. PTBD is complex as it reflects the effects of previous and persisting CKD-MBD after transplantation, the effects of immunosuppression on bone quantity and quality, and de novo CKD-MBD after transplantation [9]. In this review, we will discuss pathophysiology, risk assessment, diagnosis and management strategies to manage PTBD after kidney transplantation.

## 2. Pathophysiology

**Figure 1:**
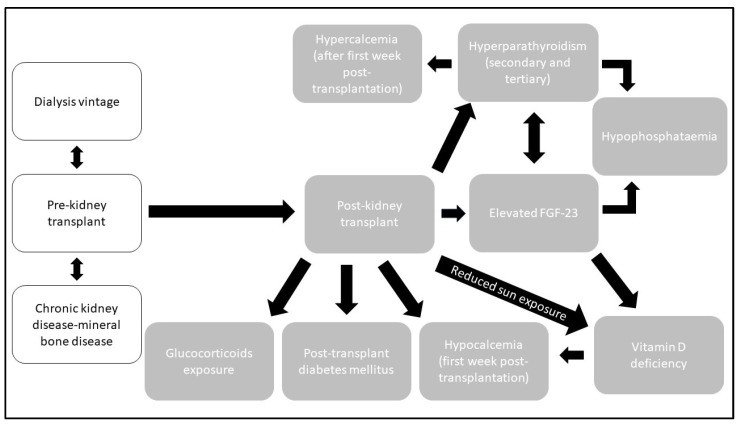
Main clinical and biochemical changes contributing to post-transplant bone disease: FGF-23, fibroblast growth factor-23.

The World Health Organization (WHO) defines osteoporosis as “a disease characterized by low bone mass and microarchitectural deterioration of bone tissue, leading to enhanced bone fragility and a consequent increase in fracture risk”. More objectively, in terms of bone mineral density (BMD) measurements, osteoporosis is defined by a value of BMD 2.5 standard deviations or more below the young adult mean [10]. The pathogenesis of osteoporosis begins prior to transplantation. Prior to kidney transplantation, patients with chronic kidney disease (CKD) or end-stage kidney disease (ESKD) on dialysis are exposed to CKD-MBD. This is then followed by post-transplantation changes in physiology influenced by medication. After transplantation, the persistence of secondary and tertiary hyperparathyroidism, renal osteodystrophy, relative choleciferol deficiency and a high level of fibroblast growth factor-23 (FGF-23) [11] contribute to PTBD. Figure 1 summarises the main pre- and post-kidney transplant contributions to PTBD.

### 2.1. Parathyroid Hormone

Elevated parathyroid hormone (PTH) is associated with increased cortical bone loss; however, the loss of trabecular bone has a U-shaped relationship with PTH [12]. In a study by Lou et al., 30.3% of patients with kidney transplantation achieved a normal PTH level within 1 year of transplantation, 26.6% between 12 and 24 months of transplantation, while 43.1% had an elevated PTH level after 2 years of transplantation [13]. Evenepoel et al. showed that post-transplantation, PTH declines in the first 3 months, followed by a slow increase up to 12 months before slowly declining again [14]. Wolf et al. found a similar trend, with a sharp PTH decline at 3 months [15]. They also found a significant positive correlation between serum PTH before and after transplantation, therefore, higher PTH level pre-transplantation carries a higher risk of persistent hyperparathyroidism following transplantation [14]. Persistent hyperparathyroidism portends an increased risk of developing PTBD [16], with a positive correlation with fracture incidence [17].

### 2.2. Calcium

Serum calcium levels fluctuate after kidney transplantation. Calcium levels decline significantly during the first week after transplantation but increase significantly thereafter. A high pre-transplantation PTH level protects against hypocalcemia within the first week but subsequently increases the risk of hypercalcemia after the first week [18]. Serum calcium levels significantly rise from 1 week post-transplant to 4 weeks and remain elevated through to 12 months post-transplantation. This is true regardless of pre-transplantation PTH level [15]. The secondary rise in serum calcium is attributed to an increase in 1,25-dihydroxycholecalciferol production in the functioning allograft post-transplantation and persistent hyperparathyroidism [7].

### 2.3. Phosphate

Serum phosphate decreases in the early phase of transplantation from week 1 to week 4, reflecting increased phosphaturia in the functioning allograft, which is stimulated by an elevated FGF-23 level. FGF-23 level decreases rapidly in the first 3 months post-transplantation and remains stable thereafter [15]. Hypophosphataemia is observed in 90% of patients in the early post-transplant period [19]. Subsequently, serum phosphate gradually increases from 3 months through to 12 months post-transplantation. There is a positive correlation between higher FGF-23 and a higher PTH level, and higher FGF-23 and a lower serum phosphate level at 3 months post-transplantation [15]. Higher FGF-23 pre-transplantation is also independently associated with low 1,25-dihydroxycholecalciferol post-transplantation [19]. An elevated PTH can lead to elevated FGF-23, which, in turn, can lead to urine phosphate wasting and accelerated bone demineralisation [20].

### 2.4. Vitamin D

25-hydroxycholecalciferol plays a pivotal role in calcium and bone homeostasis. 25-hydroxycholecalciferol deficiency can lead to hypocalcemia by reducing intestinal absorption and the kidney reabsorption of calcium, leading to the development of secondary hyperparathyroidism. 25-hydroxycholecalciferol deficiency is common after kidney transplantation. A single-centre prospective observational study by Evenepoel et al. showed that 73% of patients who received a kidney transplant were 25-hydroxycholecalciferol deficient, with 5% severely deficient at 3 months post-transplantation. Multiple factors contribute to vitamin D deficiency after transplantation. These include decreased allograft function and elevated FGF-23 and decreased PTH levels [19]. Other contributing factors include reduced sun exposure, as patients are advised to adopt sun protection measures [21], leading to reduced vitamin D absorption through the skin [20].

PTBD is distinct from CKD-BMD. This is due to the addition of other compounding factors from transplantation, including the use of glucocorticoids and immunosuppressive therapy. A study by Monier-Faugere et al. demonstrated that cumulative and maintenance doses of prednisolone and time elapsed since transplantation correlated negatively with bone volume and bone turnover [22]. Furthermore, regression analysis identified prednisolone as the main contributor to low bone volume and bone turnover. Notably, cumulative doses of cyclosporine or azathioprine, age, gender, or serum PTH did not show a negative correlation with bone volume and turnover. As previously mentioned, kidney transplantation does not reverse CKD-MBD completely. In this cohort of 57 adults living with a kidney transplant, 21% of patients were hypercalcemic, 63.2% had elevated PTH levels, and 91.2% had normal 1,25-dihydroxycholecalciferol levels. The bone turnover rate was low in 45.6% of patients, normal in 28.1% of patients and elevated in 26.3% of patients [22]. Table 1 shows the various subtypes of PTBD.

In a prospective study by Rajapakse et al., mechanical parameters, including bone stiffness and failure load of the distal tibia, were measured and compared at 2 weeks and 6 months post-kidney transplantation [24]. Bone stiffness is the resistance against deformation, and the failure load is the estimated load at which bone failure will occur [25]. Rajapakse et al. showed that both bone stiffness and failure load of the distal tibia were significantly lower 6 months post-transplantation as compared to 2 weeks post-transplantation. This study also showed that spine BMD decreased by 2.9%, while hip BMD did not change significantly on dual-energy X-ray absorptiometry (DXA) at 6 months [24]. Similar findings were reported by Iyer et al., showing that at 12 months post-transplantation, DXA did not change significantly at the spine and hip but decreased significantly in the distal radius, with similar findings at the tibia [12]. A high-resolution peripheral quantitative computed tomography (HRpQCT) of the distal radius showed a decline in both cortical and trabecular bone density, thus demonstrating that a decrease in mechanical parameters and bone density occurs in the peripheral skeleton but not the central skeleton within the first 6 and 12 months post kidney transplantation [12].

## 3. Risk Factors: Pre- and Post-Transplant Risk

### 3.1. Traditional Risk Factors

In the general population, traditional risk factors predisposing patients to osteoporosis include age, sex, low body mass index (≤19 kg/m^2^), previous fragility fracture, particularly of the hip, wrist and spine, parental history of hip fracture, current glucocorticoid treatment (any dose, by mouth for three months or more), current smoking and an alcohol intake of three or more units daily [26]. These traditional risk factors also apply to patients living with a kidney transplant [27]. In addition to organ transplantation, other secondary causes of osteoporosis, including rheumatoid arthritis, untreated hypogonadism, prolonged immobility, type 1 diabetes mellitus, gastrointestinal disease, chronic liver disease, chronic obstructive pulmonary disease, monoclonal proteinaemia, CKD with eGFR < 60 mL/min/1.73 m^2^, hyperparathyroidism, hyperthyroidism and falls, compound the risk of developing osteoporosis [26,28]. Patients living with a kidney transplant tend to be multimorbid and potentially have more than one of these secondary causes of osteoporosis.

### 3.2. Kidney-Specific Risk Factors

Patients living with CKD or kidney failure have an increased fracture rate as compared to the general population. The risk of skeletal fractures is up to 5 times higher in patients with CKD V as compared to those with CKD I and II [29]. These patients have additional risk factors exclusive to them due to the intricate interplay between kidney function and bone homeostasis. Before kidney failure ensues, CKD promotes the development of CKD-MBD, hypocalcemia, hyperparathyroidism and vitamin D deficiency, contributing to decreased bone mineral density and quality. The incidence of hip fractures is four times higher in dialysis-dependent than dialysis-independent patients [30]. In addition, Jadoul et al. showed that the adjusted odds ratio of having a hip fracture was 1.07 per 1 year longer on dialysis [31]. Thus, longer dialysis vintage prior to transplantation increases the risk of fractures in this cohort [32]. BMD losses in the early post-transplant period mainly affect cortical bones, such as the distal radius [8]. Post-transplantation risk includes the use of glucocorticoids and persistent hyperparathyroidism, which is an important risk factor for fracture [8]. In a prospective study of KTRs by Le Fur et al., a multivariate Cox analysis demonstrated an increased risk of post-transplant diabetes mellitus (PTDM) in the first-year post-transplantation in KTRs with vitamin D in the subnormal range, <30 ng/mL at the time of transplantation. KTRs with vitamin D deficiency, <10 ng/mL, had a 2.41 times higher risk for PTDM than those with normal vitamin D levels, ≥30 ng/mL [33]. Other independent risk factors of PTDM are being aged over 55 years, elevated body mass index (BMI), with a hazard ratio of 1.72 for each 5 kg/m^2^ increase, tacrolimus and maintenance corticosteroid therapy [33]. This is of interest because both vitamin D deficiency and diabetes mellitus are known risk factors for osteoporosis, which is compounded by kidney transplantation. Calcineurin inhibitor use may lead to hypomagnesaemia. Hypomagnesaemia can give rise to elevated PTH levels, resulting in increased bone turnover, thereby increasing fracture risk [34]. Evenepoel et al. showed that PTH levels did not correlate with bone changes within the first year post-transplantation, instead, the cumulative dose of corticosteroid negatively correlates with BMD at the total hip and femoral neck [35]. Therefore, whether calcineurin inhibitors affect bone turnover significantly remains unresolved.

### 3.3. Falls and Frailty

A history of falls is an independent risk factor for fracture in the general population [36], which is also applicable in a post-kidney transplant cohort. Frailty is defined as a biologic syndrome of decreased reserve and resistance to stressors resulting from cumulative declines across multiple physiologic systems, causing vulnerability to adverse outcomes [37]. CKD is associated with significant morbidity and mortality [38] and there is increasing recognition of the contribution of CKD to frailty [39]. As the population is ageing, there is a higher prevalence of frailty in people with CKD [40] compared to age-matched counterparts, compounded by the duration of CKD. Frailty is independently associated with poor outcomes, including sarcopenia, falls, cognitive impairment, and higher mortality [38]. Post-operative complications after transplant surgery, immunosuppression therapy and glucocorticoid use, which can increase the incidence of infection, malignancy, sarcopenia and osteoporosis, also contribute to frailty [41]. In a study of 59 patients living with a kidney transplant, Zanotto et al. reported that 34% of patients had at least one fall in the last 12 months, with a prevalence of falls of 1.6–4.3 times greater than the general population [42]. In a prospective cross-sectional study by Tekkarismaz et al., the risk of falling was similar between people living with kidney transplants and healthy volunteers. However, there was an increased risk of falling with elevated serum creatinine levels and allograft dysfunction [43]. McAdams-DeMarco et al. showed that patients initially become frailer during the first month before improving by 3 months post-transplantation. When frailty is coupled with a decrease in bone mineral density early post-transplantation, the risk of fractures increases with falls. However, it is worth noting that although kidney transplant recipients who were frail at the time of transplantation had higher frailty scores over the long term, they were also more likely to improve their frailty after transplantation. This supports the role of kidney transplantation as a means to reverse frailty in patients receiving dialysis therapy [44].

## 4. Risk Assessment

### 4.1. FRAX

The WHO Fracture Risk Assessment Tool (FRAX) was developed to predict the 10-year probability of hip fracture and other osteoporotic fractures based on clinical risk factors alone or in combination with bone mineral density [45]. Unfortunately, CKD or kidney failure were not included in this risk prediction model, and neither was the risk of falls [46]. However, there is accumulating evidence to suggest that FRAX is able to predict major osteoporosis in individuals with reduced kidney function. In a prospective observational study, Naylor et al. showed that the discrimination ability of FRAX to predict major osteoporotic fractures was independent of kidney function [47,48]. In another study, including patients living with a kidney transplant, the observed 10-year major osteoporotic fracture risk was concordant with FRAX prediction [49]. Hence, it would not be unreasonable to use the FRAX prediction tool to predict the risk of fracture post-transplantation to allow for early detection, modification and treatment of risk of major osteoporotic fractures.

### 4.2. DXA

DXA is a non-invasive, two-dimensional measurement of bone mineral density. It can be used in combination with FRAX to estimate 10-year probability of major fractures. Osteoporosis is defined by a BMD value of 2.5 standard deviations or more below the young adult mean [10]. However, DXA does not differentiate between cortical and trabecular bone [34,46] nor between deficit in bone volume and mineralisation [46]. DXA data may be limited and confounded by calcification of the abdominal aorta [46], which is a common complication of CKD and kidney failure [50,51]. The 2009 KDIGO Clinical Practice Guideline for the Care of Kidney Transplant Recipients suggested that patients with estimated glomerular filtration rate (eGFR) > 30 mL/min/1.73 m^2^ should have their bone mineral density measured in the first 3 months after kidney transplantation, if they receive corticosteroids or have risk factors for osteoporosis, as in the general population. However, in patients with eGFR < 30 mL/min/1.73 m^2^, BMD should not be routinely tested as BMD does not predict fracture risk as it does in the general population and does not predict the type of PTBD present [21]. That said, there is growing evidence to suggest that DXA may predict fractures in kidney transplant recipients. Akaberi et al. demonstrated that osteopenia and osteoporosis based on DXA data are both independent risk factors for fractures [52]. The predictive value of lumbar measures was less than those from the hip, likely confounded by aortic calcification [52]. The median bone mineral density value of 0.9 g/cm^2^ at the hip region was found to be a threshold value for prediction of fracture [52]. In the more recent 2017 KDIGO CKD-MBD Clinical Practice Guideline, BMD testing is suggested in all patients living with a kidney transplant across all eGFRs to assess fracture risk if results will alter therapy [8]. The frequency of DXA scanning is uncertain, however, it should not be repeated in less than 2 years and should only be repeated if the results will alter the clinical management of the patient [46].

### 4.3. Fragility Fractures

The WHO defines fragility fracture as “a fracture caused by an injury that would be insufficient to fracture normal bone; the result of reduced compressive and/or torsional strength of bone” [53]. Alternatively, fragility fracture is defined clinically as a fracture that occurs as a result of minimal trauma, a fall from a standing height or less or no identifiable trauma [54]. The risk factors for incident fragility fractures in patients living with a kidney transplant are older age, female sex, concurrent diabetes, prior fracture, receipt of pre-transplantation dialysis, dialysis vintage, glomerulonephritis and hypertension as the aetiology of kidney failure, receipt of a kidney from a deceased donor, HLA-DR mismatch and elevated urine protein-to-creatinine ratio [55]. Batteux et al. showed that in kidney transplant recipients, older age, female sex, concurrent thyroid disorders, prior fractures, especially vertebral fractures, lower serum bone-specific alkaline phosphatase, lower serum osteocalcin, lower serum PTH and lower serum creatinine were characteristics significantly associated with incident fractures [56]. Two-thirds of patients with incident fractures were shown to have osteopenia in the lumbar spine (46.3%), total hip (72.2%), or any site (47.6%), while 35.9% had osteoporosis at the wrist [56]. There is evidence showing that osteopenia and osteoporosis in the total hip BMD were associated with a significantly increased risk of fracture compared to normal BMD, independent of age, sex and diabetes status [8]. Hori et al. showed that a combination of both low muscle mass and osteoporosis increases the risk of fragility fractures (31.3%) compared with low muscle mass (11.1%) and osteoporosis (10.5%) alone [57]. Moreover, certain drug classes contribute to the risk of fragility fractures post-transplantation. The use of loop diuretics and the use of bisphosphonates were significantly associated with prevalent fractures [56]. Exposure to vitamin K antagonists, loop diuretics and opioids was significantly associated with incident fractures. Interestingly, in this study, the use of bisphosphonates and steroids was not associated with incident fractures [56]. Furthermore, exposure to loop diuretics demonstrated a significantly negative correlation with changes in T-score for the lumbar spine and wrist over time [56]. In the general population, in women over the age of 65 years with a prior fragility fracture, without knowledge of their BMD, it is cost-effective to prescribe pharmacological treatment [45]. Perhaps this should be considered in patients living with a kidney transplant with a prior fragility fracture, given the prevalence of osteopenia and osteoporosis in this cohort, especially if they were exposed to drugs associated with fragility fractures, such as loop diuretics, opioids and vitamin K antagonists.

### 4.4. Bone Biopsy

KDIGO CKD-MBD 2017 recommended considering bone biopsy to guide treatment in those within 12 months of kidney transplantation with an eGFR > 30 mL/min/1.73 m^2^ prior to commencing antiresorptive and other osteoporosis therapies [8]. However, this recommendation is not graded. Indications for bone biopsy are shown in Table 2. Bone biopsy with double-tetracycline labelling is the gold standard in accurately diagnosing the PTBD subtype [7,58], however, this is not always readily available [7]. Therefore, bone turnover markers may be used as a surrogate for bone turnover and estimating fracture risk.

### 4.5. Bone Turnover Markers

Several bone turnover markers are used to assess PTBD. PTH and bone-specific ALP (bALP) are mainly used as markers of bone turnover. In a study by Sprague et al. of patients receiving dialysis, intact PTH (iPTH) > 323.0 pg/mL (AUROC = 0.724) and bALP > 42.1 U/L (AUROC = 0.711) were used to distinguish between high and non-high bone formation rate/bone surface (BFR/BS), whereas iPTH < 103.8 pg/mL (AUROC = 0.701) and whole PTH < 48.0 pg/mL (AUROC = 0.712) and bALP < 33.1 U/L (AUROC = 0.757) were used to distinguish low from non-low BFR/BS [59]. Based on these AUROC results, iPTH and bALP may be used as guidance but were not robust enough to definitively diagnose low, normal and high bone turnover. A majority of KTRs in this study had evidence of low bone turnover renal osteodystrophy on bone biopsy in the face of an elevated PTH level. In fact, one KTR who was receiving teriparatide treatment with adynamic bone disease at baseline developed osteitis fibrosa at 6-month follow-up despite declining PTH levels [60]. Therefore, the PTH level does not reliably reflect underlying bone turnover [7], and its monitoring in KTRs has limited value in assessing PTBD [22]. In another study of patients receiving dialysis, bALP (AUROC = 0.766–0.796 with PTH > 204 pg/mL and PTH < 204 pg/mL, respectively) was used as a surrogate marker for all incident fractures [61]. A post hoc analysis of the POSTOP study in KTRs showed a negative correlation between bALP level and BMD at the hip and lumbar spine at 6 months follow-up [62]. Consequently, bALP may be a better indicator of bone turnover.

## 5. Non-Pharmacological Management

Non-pharmacological measures are recommended in all patients living with a kidney transplant, especially in those with increased risk of PTBD and fractures. The general recommendations for the management of osteoporosis for the general population should be applied. The National Osteoporosis Guideline Group strongly recommends a healthy, nutrient-rich balanced diet with an adequate intake of calcium (minimum 700 mg daily), preferably through diet or otherwise via supplementation, and to consume vitamin D from food or otherwise supplementation of at least 800 IU/day if they are vitamin D deficient or at risk of deficiency. Calcium and vitamin D supplementation is described further in the next section. Moreover, alcohol intake should be restricted to ≤2 units daily and smoking cessation is recommended [26]. Low BMI, sarcopenia and low bone mass are associated with increased fracture risk and fragility fractures [63]. Thus, tailored regular weight-bearing exercises and muscle-strengthening exercises should be encouraged among those at risk of osteoporosis to build muscle mass and strength and to improve balance. A formal fall assessment should be conducted in all patients with osteoporosis [26], including a medication review to eliminate medications that could contribute to fall risk. Introducing pragmatic measures to counter falls, including improving senses (hearing aids and glasses), adequate lighting when mobilising and appropriate footwear to improve grip, are advised [63].

Prevention by means of screening can be beneficial pre-transplantation in terms of improving post-transplantation outcomes in regard to bone health and preventing the development of PTBD. Risk factor identification, including traditional risk factors, kidney-specific risk factors, fall risk and frailty, as described in the previous section, should be incorporated as part of the pre-transplantation work-up. Any risk identified should be addressed early, and the non-pharmacological measures listed above should be encouraged and adopted by patients awaiting kidney transplants. Early mobilisation and continuous engagement with exercise, especially weight-bearing exercises, should be implemented after kidney transplantation to prevent loss of muscle mass and bone mass during the post-transplantation period.

## 6. Pharmacological Management

### 6.1. Glucocorticoid Minimisation

Cumulative dose of corticosteroid negatively correlates with trabecular bone volume and bone turnover but shows no effect on the mineralisation status [22]. Thus, minimising glucocorticoid exposure after transplantation reduces the negative impact of corticosteroids on trabecular bone volume and bone turnover [64]. Nikkel et al. demonstrated a 31% reduction in fracture risk in patients managed with early corticosteroid withdrawal (ECSW) compared to corticosteroid-based immune suppression (CSBI) by 24 months after transplantation based on USRDS data [65]. On the contrary, Vautour et al. showed that cumulative corticosteroid dose was not associated with increased fracture risk [66]. Interestingly, in a prospective study by Woodle et al. with a median follow-up of 15.8 years after kidney transplantation, there was no significant difference in allograft failure from any cause, including death between ECSW and CSBI regime [67]. Given this, consideration could be given to ECSW over CSBI regimen post-transplantation to reduce exposure to corticosteroids in those who are at high risk of fractures to minimise the risk of corticosteroid-associated adverse events. Nevertheless, this has to be balanced with the risk of acute allograft rejections and allograft loss identified in other studies. In a large retrospective study of 5170 KTRs in Austria in 2017, Haller et al. found significantly more acute rejections in KTRs where glucocorticoids were discontinued within the first 12 months of transplantation [68]. In KTRs who had glucocorticoid withdrawal within 18 months post-transplantation, there were significantly higher rates of allograft loss, which was not observed when glucocorticoids were withdrawn after 2 years of transplantation [68]. Ekberg et al. demonstrated similar incidences of PTDM within the first year post-transplantation between glucocorticoid avoidance and glucocorticoid maintenance group of KTRs. This similarity persisted at 2 years post-transplantation. In this study, the incidence of biopsy-proven rejections were numerically higher in the glucocorticoid avoidance group but not statistically different from the glucocorticoid maintenance group [69]. As there is conflicting evidence advocating for and against glucocorticoid avoidance in KTRs, personalised risk should be taken into consideration when deciding on a glucocorticoid regimen post-transplantation, with reference to the risk of PTBD, PTDM, allograft rejection and allograft loss.

Treatment with cholecalciferol, 1,25-dihydroxycholecalciferol/alfacalcidol and/or antiresorptive agents was suggested in patients in the first 12 months after kidney transplantation with an eGFR > 30 mL/min/1.73 m^2^ in the KDIGO 2017 CKD-MBD guidelines. Treatment choices should be guided by the subtype of CKD-MBD using serum calcium, phosphate, PTH, alkaline phosphatase and vitamin D levels as biomarkers. However, it is worth noting that KDIGO 2017 guidelines highlight that there are insufficient data to guide the treatment of bone disease after the first 12 months post-transplantation [8]. Table 3 shows a summary of various pharmacological management for PTBD.

### 6.2. Calcium

The recommended daily dietary allowance for calcium is between 1000 and 1200 mg based on age and sex [70]. Calcium supplementation in patients with kidney failure is complex, given the altered bone, calcium, phosphate and PTH homeostasis. Elevated serum calcium has been associated with non-fatal cardiovascular events. Therefore, patients living with a kidney transplant should adhere to the recommended dietary allowance, ideally via dietary intake, if not, via supplementation, to maintain calcium levels within the normal range while avoiding hypercalcemia [8]. The treatment of hypocalcemia can reduce the risk of secondary hyperparathyroidism and improve BMD in combination with vitamin D [63]. In a systematic review by Palmer et al., there was no difference in the risk of hypercalcemia when treated with both calcium and vitamin D than calcium alone in KTRs [71].

### 6.3. Vitamin D

The recommended daily dietary allowance for vitamin D is between 600 and 800 IU, depending on age and sex [70]. Vitamin D deficiency is common post-transplantation. Sadlier et al. reported in a transplant cohort that a majority (88%) of patients had abnormal vitamin D levels during the first year post-transplantation; 29% had vitamin D deficiency, and 59% had vitamin D insufficiency [72]. A more recent study by Tsujita et al. showed that 96% of patients living with a kidney transplant had vitamin D deficiency one-month post-living donor kidney transplantation [73]. Vitamin D treatment reduces the rate of persistent hyperparathyroidism at 1 year post-transplantation from 39% to 25% [17]. Treatment with vitamin D shows contradictory results for BMD outcomes post renal transplant. In a systematic review by Palmer et al., vitamin D treatment showed no benefit on BMD at the lumbar spine’ however, some studies have shown that treatment with cholecalciferol 25,000 IU as a single monthly dose was associated with reduced BMD at the lumbar spine [71,74]. Recently, a prespecified secondary endpoint analysis of a double-blind placebo-controlled randomised controlled trial conducted by Tsujita et al. reported that treatment with cholecalciferol 4000 IU/day versus placebo significantly increased vitamin D levels and significantly reduced PTH levels at 12 months post-transplantation, with greater treatment effect in those with higher eGFR, lower calcium and lower vitamin D levels [73]. Furthermore, there was a significantly lower mean percent change in lumbar spine BMD in the cholecalciferol-treated group (−0.2%) versus placebo (−1.9%) [73]. In addition, this beneficial effect on lumbar spine BMD was more prominent in those with lower baseline lumbar spine BMD. In patients with osteopenia or osteoporosis at the lumbar spine at baseline, there was an increase in BMD after 11 months of treatment with cholecalciferol 4000 IU/day, but not in those with normal BMD at baseline [73]. There was no difference between both groups in percent change in BMD at the distal radius and incidence of hypercalcemia [73]. A mediation analysis of this study showed that the change in whole PTH level explained 40% of the treatment effect on lumbar spine BMD. It is worth noting that this study was conducted in Japanese patients living with a kidney transplant, recruited 1 month post-living donor kidney transplant and maintained on CSBI, with a cumulative mean dose of prednisolone of 2981 mg in the cholecalciferol group and 2920 mg in the placebo group [73]. Given this new evidence, treatment with cholecalciferol after kidney transplantation should be especially targeted in those with osteopenia or osteoporosis on BMD, vitamin D deficiency and/or elevated PTH level.

### 6.4. Vitamin D Receptor Activators (VDRAs)

A high bone turnover state in persistent secondary hyperparathyroidism post-transplantation is driven by an elevated PTH level [75]. VDRAs, such as alfacalcidol, calcitriol and paricalcitol, can lower PTH levels and improve BMD after transplantation [71,76]. Treatment with paricalcitol has been shown to significantly lower bone turnover markers, such as bALP and osteocalcin levels, within 3 months of therapy [75]. Although paricalcitol has been associated with a higher risk of hypercalcemia [76], Trillini et al. reported no increase in serum calcium, phosphate and vitamin D levels. Overall, paricalcitol showed an increase in lumbar spine BMD at 6 months [75]. A systematic review showed that combination therapy of calcitriol and calcium significantly improved BMD at the lumbar spine and femoral neck when compared to placebo or no treatment [71].

### 6.5. Bisphosphonates

Bisphosphonates are analogues of inorganic pyrophosphate. They bind to hydroxyapatite crystals with high affinity for bone minerals. They inhibit hydroxyapatite breakdown, thus suppressing bone resorption [77]. A systematic review by Palmer et al. showed that bisphosphonate therapy in a kidney transplantation cohort significantly improved BMD by DXA at the lumbar spine and femoral neck when compared to placebo or no treatment, but there was no difference in fracture rates [71]. More recently, Hauck et al. reported a 15-year single-centre real-world experience study in KTRs, in whom there was a significant decline in lumbar spine BMD in patients not treated with bisphosphonates (−0.064 ± 0.050 g/cm^2^) compared to a significant increase in BMD when treated with bisphosphonates (0.054 ± 0.055 g/cm^2^, *p* = 0.001). At the hip, there was a non-significant decline in BMD in both bisphosphate-treated and non-treated groups, however, numerically, the rate of decline was lower in the group receiving bisphosphonates [78]. When bisphosphonates were compared to alfacalcidol and calcitriol, bisphosphonates showed significant benefits in terms of BMD at the lumbar spine and the femoral neck. No significant effect of treatment with bisphosphonates for risk of low bone turnover and hypocalcemia was observed [71]. The use of bisphosphonates in patients living with kidney transplants did not result in a difference in ALP and PTH levels before and after treatment and had no adverse effects on eGFR [78]. Bisphosphonates, when not retained in the bone, are excreted by the kidneys [77]. Thus, their excretion can be reduced in cases of impaired kidney function. Amerling et al. showed a risk of adynamic bone disease onbone biopsy with the use of bisphosphonates in a cohort of patients with CKD II–IV [79]. Bisphosphonates can cause adverse events such as osteonecrosis of the jaw, atypical femoral fracture and severe hypocalcemia [58]. Therefore, bisphosphonate use should be considered in those with a high risk of fracture but should be used with caution, especially in those who may have adynamic bone disease.

### 6.6. Denosumab

Denosumab is a fully human IgG2 monoclonal antibody that binds human receptor activator of NF-κB ligand (RANKL) with high affinity. Its use reduces osteoclast numbers and bone turnover, resulting in a significant increase in bone mass and density at the spine and hip. Moreover, cortical sites, including the femoral shaft, benefit from denosumab therapy, with increased strength due to an increase in cortical thickness and/or reduced cortical porosity [80]. Bonani et al. reported a prospective study of patients receiving a kidney transplant within 28 days maintained on CSBI, in whom denosumab therapy every 6 months in the first year after kidney transplantation was associated with increased areal BMD at the lumbar spine, hip and femoral neck. Denosumab also increased volumetric BMD at the distal tibia and radius. Subgroup analysis in this study showed that the effect of improved lumbar spine areal BMD at 12 months was stronger in patients who were male and younger, with lower T-scores, higher eGFR and lower PTH levels at baseline [81]. Other studies have also demonstrated significant improvements in vertebral and hip BMD with denosumab therapy in patients living with kidney transplants [82,83]. However, these studies are limited by small cohort sizes and short duration of follow-up, therefore, data regarding fracture incidence was not reported. In the FREEDOM study, denosumab therapy was associated with both an increase in BMD at the lumbar spine and total hip and a reduction in the risk of vertebral, nonvertebral, and hip fracture in postmenopausal women with osteoporosis [84]. A post hoc analysis of the FREEDOM study showed that increased BMD and reduced fracture risk did not differ in CKD I to CKD IV [85]. Thus, it may be reasonable to predict that improvement in BMD with denosumab therapy in patients living with a kidney transplant should improve their fracture risk. The use of denosumab in KTRs may be associated with significantly higher rates of urinary tract infections and diarrhoea compared to placebo [81]. Bonani et al. also reported more frequent asymptomatic and transient hypocalcemia in the denosumab-treated group [81], while Alfieri et al. reported a significant decline in serum calcium from 9.6 mg/dL (2.4 mmol/L) to 9.4 mg/dL (2.35 mmol/L) in 1 year, although no symptomatic or asymptomatic hypocalcemia was reported [83]. Recently, a retrospective study in Taiwan by Tsai et al. showed a significant increase in hypocalcemia in denosumab-treated KTRs with eGFR < 45 mL/min/1.73 m^2^ compared to other antiresorptive therapies [86]. Hypocalcemia tends to occur in those with more advanced renal impairment (CKD IV–V) in the first week after denosumab treatment [82]. Hence, calcium and vitamin D levels should be monitored before and within 1 month of treatment with denosumab to prevent and allow for prompt treatment of hypocalcemia. Hypocalcemia, when detected, should be treated with increased calcium supplementation [82,83]. There is also a risk of rebound bone remodelling when denosumab is ceased, leading to decreased cortical thickness and trabecular bone volume and increased unmineralised bone due to the rapid acceleration of bone turnover [87]. Thus, the cessation of denosumab therapy must be performed in a controlled fashion, with a background of alternative medication to mitigate this rebound effect.

### 6.7. Teriparatide

Teriparatide is a recombinant peptide of the first 34 amino-terminal residues of PTH. It is an anabolic agent used in the treatment of osteoporosis to stimulate bone formation, resulting in a lower risk of vertebral and nonvertebral fractures and an increase in vertebral, femoral and total body BMD [88]. A post hoc analysis of a randomised controlled trial of 1637 postmenopausal women with a median follow-up duration of 21 months revealed a lower incidence of vertebral and nonvertebral fractures in the teriparatide-treated group when compared to placebo, with similar benefits in the group with normal and impaired kidney function. Treatment with 20 mcg/day and 40 mcg/day of teriparatide therapy in participants with impaired kidney function resulted in a significant increase in mean BMD at the lumbar spine and femoral neck at 18 months and 12 months, respectively. This finding was statistically significant across renal function subgroups. Adverse events associated with impaired kidney function were hypercalcemia and hyperuricemia, which were reported to be dose-dependent and more severe in those with lower eGFR [89]. A double-blind randomised controlled trial with a 6-month follow-up completed in 24 KTRs by Cejka et al. showed that femoral BMD remained stable with 20 mcg/day teriparatide. However, those receiving placebo had a significant decline in their femoral BMD [60]. Despite evidence of its use in CKD, KTRs treated with teriparatide in this study did not show any changes in BMD at the lumbar spine and radius, histomorphometric bone volume or bone matrix mineralisation status. Among the 12 patients who received bone biopsy at the time of kidney transplantation, eight (66.%) had adynamic bone disease, three (25%) had mixed uraemic bone disease and only one (8.3%) had a normal bone biopsy, despite concurrent elevated PTH levels [60]. This is suggestive of post-transplant PTH resistance, which may explain the lack of positive findings in this study. However, it is worth acknowledging that this study was conducted in a small cohort over a 6-month period, so it may not have been powered to demonstrate the effect of teriparatide in KTRs.

### 6.8. Calcimimetics

Calcimimetics are positive allosteric modulators of the calcium-sensing receptor that increase the sensitivity of the parathyroid glands to circulating calcium. They also increase vitamin D receptor expression, encouraging the use of concurrent vitamin D for the management of secondary hyperparathyroidism [90]. Cinacalcet has not been approved for use in patients living with a kidney transplant for the treatment of hyperparathyroidism [8]. However, studies have shown the safe and efficacious use of cinacalcet for treating hypercalcemia in secondary hyperparathyroidism, resulting in a significant reduction in serum calcium and a rise in serum phosphate among KTRs [91,92]. In a randomised controlled trial by Evenepoel et al., there was no significant change in BMD at the femoral neck between cinacalcet and placebo [93], similar to findings in a non-transplant population [94]. Since persistent secondary hyperparathyroidism is associated with increased fracture incidence, one might assume that treating it would reduce fracture incidence. However, there are conflicting results in regard to the effect of cinacalcet on serum ALP, a biomarker sometimes used as a marker of bone turnover [95].

**Table 3:** Summary of pharmacological management of PTBD.

Pharmacological Therapy	Study	Results/Recommendations
Glucocorticoid minimisation	Nikkel et al. [65]	31% reduction in fracture risk with ECSW than CSBI by 24 months after transplantation
	Vautour et al. [66]	Cumulative corticosteroid dose was not associated with increased fracture risk
Calcium	Ross et al. [70]	Recommended dietary allowance for calcium is between 1000 and 1200 mg daily based on age and sex
	KDIGO 2017 CKD-MBD [8]	If recommended dietary allowance is not met, then oral supplementation is recommended to maintain calcium within a normal range
	Haarhous et al. [63]	Treatment of hypocalcemia can reduce the risk of secondary hyperparathyroidism and improve BMD in combination with vitamin D
Vitamin D	Ross et al. [70]	Recommended daily dietary allowance for vitamin D is between 600 and 800 IU, depending on age and sex
	Perrin et al. [17]	Reduces the rate of persistent hyperparathyroidism at 1 year post-transplantation from 39% to 25%
	Palmer et al. [71]	Showed no benefit on BMD in the lumbar spine
	Tsujita et al. [73]	Treatment with cholecalciferol 4000 IU/day versus placebo significantly increased vitamin D level, and significantly reduced PTH level at 12 months post-transplantation, with greater treatment effect in those with higher eGFR, low calcium and low vitamin D level Significantly lower mean percent change in lumbar spine BMD in the cholecalciferol-treated group (−0.2%) versus placebo (−1.9%) In patients with osteopenia or osteoporosis in the lumbar spine at baseline, there was an increase in BMD after 11 months of treatment with cholecalciferol 4000 IU/day, but not in those with normal BMD at baseline
VDRA	Trillini et al. [75]	Treatment with paricalcitol significantly lowers bone turnover markers such as bALP and osteocalcin levels within 3 months of therapy and increases lumbar spine BMD at 6 months
	Palmer et al. [71]	Combination therapy of calcitriol and calcium significantly improved BMD at the lumbar spine and femoral neck when compared to placebo or no treatment
Bisphosphonates	Palmer et al. [71]	Bisphosphonate therapy in a kidney transplantation cohort significantly improved BMD by DXA at the lumbar spine and femoral neck when compared to placebo or no treatment, but no difference in fracture rates Bisphosphonates showed significant benefits for BMD at the lumbar spine and the femoral neck when compared to alfacalcidol and calcitriol
	Hauck et al. [78]	Bisphosphonates therapy resulted in a significant increase in BMD while non-treated group had a significant decline in lumbar spine BMD
	Amerling et al. [79]	Risk of adynamic bone disease, on bone biopsy with use of bisphosphonates in a cohort of patients with CKD II–IV
Denosumab	Bonani et al. [81]	Denosumab 6-monthly used in the first year after transplantation resulted in increased areal BMD at the lumbar spine, hip and femoral neck, and increased volumetric BMD at the distal tibia and radius
	Brunova et al. [82], Alfieri at al. [83]	Denosumab therapy in patients living with kidney transplant resulted in a significant improvement in vertebral and hip BMD
	Jamal et al. [85]	Denosumab therapy led to increase in BMD and reduction in fracture risk, which did not differ in CKD I to CKD IV
Teriparatide	Miller et al. [89]	Treatment with 20 mcg/day and 40 mcg/day of teriparatide therapy in participants with impaired kidney function resulted in a significant increase in mean BMD in the lumbar spine and femoral neck at 18 months and 12 months, respectively
	Cejka et al. [60]	Femoral BMD remained stable with 20 mcg/day teriparatide while those receiving placebo had significant decline in their femoral BMD
Calcimimetics	Alpay et al. [91], Zavvos et al. [92]	Significant reduction in serum calcium and a rise in serum phosphate among KTRs when cinacalcet is used to treat hypercalcemia in secondary hyperparathyroidism,
	Evenepoel et al. [93]	No significant change in BMD at the femoral neck between cinacalcet and placebo

## 7. Conclusions

PTBD is complex and multifaceted owing to the development of CKD-MBD prior to kidney transplantation, followed by the additional insult to bone integrity when on dialysis, and finally, acquired bone disease after transplantation due to changes in physiology induced by allograft and immunosuppression. It includes but is not limited to, the development of hypocalcemia, hypercalcemia, hypomagnesaemia, hypophosphataemia, vitamin D deficiency, hyperparathyroidism, osteomalacia, osteopenia and osteoporosis. There is a degree of diagnostic uncertainty in PTBD due to the lack of validated and readily available biomarkers in this population. The gold standard to diagnose PTBD is to perform a bone biopsy. However, bone biopsy is not readily available, is time-consuming and has associated risks. There is a growing body of evidence to support the use of other biomarkers as surrogates. There are multiple tools available that can be used to help decision making in terms of diagnosis and management. These include the FRAX score for fracture risk, an assessment of BMD using DXA and bone turnover markers. Ideally, optimal calcium and vitamin D intake should be achieved with dietary measures in the first instance. However, calcium and vitamin D supplementation should be prescribed in those with calcium and vitamin D deficiency. VDRA can be used to lower PTH levels and bone turnover markers in conjunction with BMD management. It is reasonable to consider the use of bisphosphonates and denosumab in KTRs with clinical and biochemical evidence of high bone turnover to maintain bone quality and quantity in order to reduce fracture risk. However, there is a risk of precipitating adynamic bone disease in KTRs. Therefore, if the diagnosis is uncertain, treatment with these agents should be avoided until a definitive diagnosis is obtained. In the event of low bone turnover, teriparatide could be considered to prevent further loss of BMD. Moreover, a multidisciplinary approach is often necessary when dealing with complex patients with pathology, such as PTBD in KTRs, involving nephrology, endocrinology, rheumatology, radiology and pathology. Shared decision making between clinicians and patients should be adopted when starting any of these agents, taking into account the risk–benefit evaluation and patients’ acceptance of risk.

Future research in this area should focus on the evaluation of current existing therapies beyond the first year post-transplant, development of biomarkers or metabolite signatures to identify and diagnose PTBD subtype and research into biomarkers to monitor the efficacy of a treatment.

## Figures and Tables

**Table 1:** Classification of renal osteodystrophy.

Post-Transplant Bone Disease Including Renal Osteodystrophy [23]
Osteitis fibrosa/advanced hyperparathyroidism-related bone disease (high bone turnover)
Adynamic bone disease (low bone turnover)
Osteomalacia (low bone turnover)
Mixed renal osteodystrophy (either high or low bone turnover)
Osteopenia and osteoporosis *

* Not specific to kidney transplant recipients (KTRs).

**Table 2:** Indication of bone biopsy in patients with CKD [23].

Indications for Bone Biopsy
Inconsistencies among biochemical parameters precluding a definitive diagnosis
Unexplained skeletal fracture or bone pain
Severe progressive vascular calcification
Suspicion of overload or toxicity from aluminum or other metals
Before parathyroidectomy for advanced secondary or tertiary hyperparathyroidism
Before commencing bisphosphonates
